# Socioeconomic factors associated with diarrheal diseases among under-five children of the nomadic population in northeast Ethiopia

**DOI:** 10.1186/s41182-016-0040-7

**Published:** 2016-12-09

**Authors:** Wondwoson Woldu, Bikes Destaw Bitew, Zemichael Gizaw

**Affiliations:** 1 Hadaleala District Health Office, Hadaleala District, Afar Regional State Ethiopia; 2 Department of Environmental and Occupational Health and Safety, University of Gondar, Gondar, Ethiopia

**Keywords:** Childhood diarrhea, Under-five children, Socioeconomic factors, Nomads, Afar Region

## Abstract

**Background:**

Diarrheal disease remains the leading cause of morbidity and mortality among under-five children worldwide. Every day, more than 4000 children lose their lives due to diarrhea. In Ethiopia, diarrhea is the second killer of under-five children next to pneumonia.

**Methods:**

A cross-sectional study was conducted to assess the prevalence of under-five diarrhea and socioeconomic factors among the nomadic people in Hadaleala District. A total of 704 under-five children were included in this study, and subjects were recruited by the multistage cluster sampling technique. Data were collected by a pre-tested questionnaire. The multivariable logistic regression analysis was used to identify socioeconomic variables associated with childhood diarrhea.

**Results:**

The 2-week period prevalence of diarrhea among under-five children was 26.1% (95% CI 22.9, 29.3%). The highest prevalence (37.5%) of diarrhea occurred among children aged between 12.0 and 23.0 months. The occurrence of diarrheal disease was associated with the presence of two (AOR = 4.3, *p* < 0.001) and three (AOR = 22.4, *p* < 0.001) under-five children in each household. The age of the children ranged between 6.0 and 11.0 months (AOR = 4.8, p < 0.001), 12.0 and 23.0 months (AOR = 6.0, *p* < 0.001), and 24.0 and 35.0 months (AOR = 2.5, *p* < 0.05), illiterate mothers (AOR = 2.5, *p* < 0.05), and poor households (AOR = 1.6, *p* < 0.05).

**Conclusions:**

Diarrhea prevalence was quite high among under-five children in Hadaleala District, and it was significantly concentrated among children aged between 12.0 and 23.0 months. The number of under-five children, age of children, mothers’ education, and household economic status were significantly associated with childhood diarrhea. To minimize the magnitude of childhood diarrhea, implementing various prevention strategies such as health education, child care, personal hygiene, and household sanitation which can be integrated with the existing national health extension program are essential.

## Background

In 2010, 7.6 million under-five children died worldwide, nearly 21,000 under-five children every day. The highest rates of child mortality are seen in sub-Saharan Africa, where 1 in 8 children dies before age 5, more than 17 times the average for developed regions. Each year, 1.5 million deaths occur due to diarrheal disease [1]. Diarrheal disease remains the leading cause of morbidity and mortality in children under 5 years of age. Every day, more than 4000 children lose their life due to diarrhea [2]. The vast majority of these deaths are among children who live in low- and middle-income countries [3].

In Ethiopia, diarrheal disease is a major public health problem. The 2010 report of the Ministry of Finance and Economic Development (MOFED) indicated that 20% of childhood deaths in the country were due to diarrhea. The 2011 Demographic and Health Survey of Ethiopia (EDHS) findings also showed that 13% of the children had diarrhea in the 2 weeks preceding the survey at the national level [4, 5].

The Afar Region is one of the poorest, least developed, and under-serviced regions of Ethiopia and has the highest child mortality rate. It is estimated that 6459 children under the age of five still die each year, and this mortality rate is 123/1000 live births [6]. The communities that live in Hadaleala District are nomads. Nomads migrate mostly in search of pasture and water. The community has been suffering from shortages of water, hygiene, and sanitation facilities. The main sources of water for the community are river, streams, ponds, and wells that provide water for domestic use and for animals. During 2015, safe water and sanitation coverage of the district was 35 and 12%, respectively [7]. The community depends on livestock as a major subsistence economic activity, based on traditional pastoralist systems tending camels, goats, cattle, sheep, and donkeys [7]. All these situations are possible risk factors for the occurrence of mainly childhood diarrheal disease and other water-, hygiene-, and sanitation-related communicable diseases.

Childhood diarrhea has resulted from interactions of socioeconomic factors. Literatures show that the education status of family members, occupational status of mothers and fathers, family size, number of under-five children, household economic status, age of children, and other socioeconomic factors contribute to diarrheal disease. According to the literatures, socioeconomic factors have a role in the occurrence of communicable diseases through their indirect link with the quality of life, access to healthcare facilities, access to adequate water and environmental sanitation, the opportunity to use different hygienic methods, and awareness and behavior relating to disease prevention [5, 8–14]. Though the health burden of diarrheal diseases is widely recognized at the global level, there is limited information on its prevalence and the socioeconomic factors contributing to its occurrence among the nomadic population of Ethiopia. This study was therefore designed to assess the prevalence of under-five diarrheal disease and socioeconomic factors among nomadic people in Hadaleala District, Afar Region, northeast Ethiopia. The result of this study could help the national, regional, and zonal level policy makers, health institutions at each level, and the community to design and implement strategies to prevent or minimize childhood diarrheal disease. Furthermore, it may also serve as a baseline data for further studies and local consumption.

## Methods

### Study design and settings

A community-based cross-sectional study was conducted among the nomadic populations in Hadaleala District, Afar Region, northeast Ethiopia in May, 2015. Hadaleala District is one of the districts of Hariresu Zone, Afar National Regional State. It is located at 341 km southwest of the regional capital, Semera, and 268 km north of Addis Ababa, the capital city of Ethiopia. It has an area of 1272 km^2^ divided into 11 rural kebeles (the smallest administrative units in Ethiopia) with a total population of 42,845 as projected for the year 2015. It has 7516 households with an average household size of 5.7 persons per house. Under-five children account for 10.1% (4328) of the total population. As the population lives in a very scattered manner, the average population density is 14 persons/km^2^_._ Furthermore, the economy of the district is based on livestock and crop production [7]. Due to the dispersed pasture and water resources, the nomadic communities in the district are mobile.

### Sample size determination

The sample size was determined using the single population proportion formula by considering the following assumptions: *p* = 31.0% (the 2-week period prevalence of diarrhea among under-five children in Arba Minch District) [9], 95% confidence interval, and a 5% margin of error (d),

$$ n=\frac{{\left({z}_{\raisebox{1ex}{$\alpha $}\!\left/ \!\raisebox{-1ex}{$2$}\right.}\right)}^2p\left(1-p\right)}{d^2}=\frac{(1.96)^20.31\left(1-0.31\right)}{0.05^2}=328 $$

Considering the design effect of 2 and 5% non-response rate, the final sample size was 689 mother–child pair.

### Sampling procedure

The multistage cluster sampling technique was used to select study participants from the nomadic population. The clusters were villages with defined geographical boundaries. Out of a total of 11 kebeles, 6 were selected by the simple random sampling technique. The 6 selected kebeles were clustered into 39 villages, and 17 villages were selected by the systematic random sampling technique. Finally, all households (704) with under-five children were included in the study. For households which had more than one child each, the younger one was selected for the study.

### Measurement of outcome variable

Childhood diarrheal disease, the primary outcome variable of this study, is defined as having three or more loose or watery stools in 24 h [15, 16]. The prevalence of childhood diarrheal disease within the 2-week period prior to data collection was calculated as the total number of diarrhea cases divided by 704 (the total number of under-five children participating in the study). Household economic status was also calculated by using tropical livestock unit (TLU) [17]. During the survey, the number and species of livestock were assessed. The main categories of domestic livestock included in this study were large ruminants (cattle and camels), small ruminants (sheep and goats), non-ruminant grazing animals (asses, mules, and horses collectively known as equines), and chickens. To determine the household economic status in relation to domestic livestock, the TLU conversion factors were used (Table 1). TLU was determined as (1.0 × number of camels) + (0.7 × number of cattle) + (0.1 × number of sheep) + (0.1 × number of goats) + (0.8 × number of horses) + (0.7 × number of mules) + (0.5 × number of asses) + (0.01 × number of chickens). A below 5 TLU score indicated that the household was poor. A TLU score of 5 to 12.99 showed the household was medium in economic status, and rich households scored 13 and above TLU [18].

**Table 1:** Species of domestic livestock and TLU conversion factors to determine households’ economic status in Hadaleala District, Afar Region, northeast Ethiopia, May, 2015

Species	TLU conversion factors
Camels	1.0
Cattle	0.7
Sheep	0.1
Goats	0.1
Horses	0.8
Mules	0.7
Asses	0.5
Chicken	0.01

### Data collection tools and procedures

A pre-tested structured questionnaire was used to collect data. The questionnaire was prepared in English and translated to the local language and back translated to English to maintain the consistency of the questions. The tool was pre-tested out of the study area in a community which had similar characteristics prior to the actual data collection. To improve the quality of the data, eight diploma graduate nurses and two environmental health officers who were fluent enough in both Amharic and Afarigna (local languages) and working in the district were involved in the data collection process. After the pretest and training of data collectors, the data collectors visited all households in the selected clusters. When the data collectors found under-five children during the visits, they interviewed the mothers about the variables. The youngest (at the time of the survey) children were included in the study when there were more than one under-five children in the household. Finally, the collected data were checked and corrected by the data collectors immediately after finalizing the questionnaire. Supervisors daily checked the completeness, quality, and consistency of information collected, and the correctness of the information was checked by recollecting data from 5% of the households which provided the original data.

### Data management and statistical analysis

Data were entered using the EPI-INFO version 3.5.3 statistical package and exported to SPSS version 20 for further analysis. Cross tabulation was used to describe socioeconomic characteristics and childhood diarrhea. Categorical data were presented as frequency counts or percentages and compared using the Pearson chi-square. Continuous data were summarized as means or medians with ± standard deviations and interquartile ranges. The univariable logistic regression analysis was used to choose variables for the multivariable logistic regression analysis, and variables which had less than 0.2 *p* values by the univariable analysis were then analyzed by the multivariable logistic regression for controlling the possible effects of confounders.

## Results

### Socioeconomic characteristics of respondents

A total of 704 under-five children and their mothers participated in the study with a 100% response rate. Nearly one third, 229 (32.5%), of the children were aged above 35.0 months. The median age of the children was 24.0 months, and the interquartile range (IQR) was 11.0–38.0 months. The majority, 425 (60.4%), of the households had only one child, and more than half, 378 (53.7%), of the children were male. More than half, 362 (51.4%), of the mothers were aged between 25.0 and 34.0 years. The median age of the mothers was 29.0 years, and the IQR was 24.0–43.0 years. The great majority, 687 (97.6%), of the mothers were currently engaged. Six hundred thirty-three (89.9%) mothers were Afar by ethnicity. Six hundred twenty-four (88.6%) mothers had no formal education. The majority, 668 (94.9%), of the mothers were housewives by occupation. About 456 (64.8%) households were economically poor. More than half, 371 (52.7%), of the households had more than five family members (Table 2).

**Table 2:** Socioeconomic information of households (*n* = 704) in Hadaleala District, Afar Region, northeast Ethiopia, May, 2015

Variables	Frequency	Percent
Number of under-five children in the house		
One	425	60.4
Two	253	35.9
Three	26	3.7
Age of children in months		
<6.0	113	16.1
6.0–11.0	72	10.2
12.0–23.0	152	21.6
24.0–35.0	138	19.6
>35.0	229	32.5
Sex of children		
Male	378	53.7
Female	326	46.3
Age of mothers		
15.0–24.0	178	25.3
25.0–34.0	362	51.4
≥35.0	164	23.3
Marital status of mothers		
Currently engaged	687	97.6
Currently not engaged	17	2.4
Ethnicity of mothers		
Afar	633	89.9
Oromo	56	8.0
Amhara	15	2.1
Educational status of mothers		
No formal education	624	88.6
Formal education	80	11.4
Occupational status of mothers		
Housewife	668	94.9
Employed	36	5.1
Household livestock ownership		
Yes	687	97.6
No	17	2.4
Economic status of households		
Poor	456	64.8
Medium	248	35.2

### Prevalence of diarrheal disease among under-five children

**Fig. 1:**
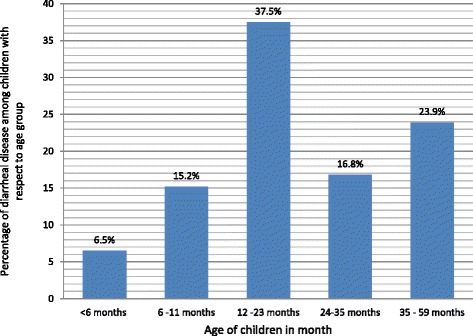
Diarrheal cases with respect to age among under-five children in Hadaleala District, Afar Region, northeast Ethiopia, May, 2015

A total of 184 children had diarrhea in the 2-week period prior to data collection. Therefore, the 2-week period prevalence of diarrhea among under-five children was found to be 26.1% (95% CI 22.9, 29.3%). Moreover, 81 children had diarrhea at the time of data collection, and therefore, the point prevalence was found to be 11.5% (95% CI 9.1, 13.8%). The highest prevalence of diarrhea, 69 (37.5%), occurred among children aged 12.0–23.0 months (Fig. 1). Sixty-one (8.7%) of the mothers reported that they had diarrhea in the 2 weeks preceding the survey. More than half, 102 (55.4%), of the children who had diarrhea obtained treatment from public health facilities (Table 3).

**Table 3:** Occurrence of diarrheal disease among under-five children (*n* = 704) and their mothers and measures taken in Hadaleala District, Afar Region, northeast Ethiopia, May, 2015

Variables	Frequency	Percent
Two-week period childhood diarrhea		
Yes	184	26.1
No	520	73.9
Under-five diarrhea at the time of data collection		
Yes	81	11.5
No	623	88.5
Mothers’ diarrhea in the 2-week period		
Yes	61	8.7
No	643	91.3
Measures taken for childhood diarrhea		
Treatment from health institution	102	55.4
Home medication	82	44.6
Measures taken for mothers’ diarrhea		
Treatment from health institution	6	9.8
Home medication	55	90.2

### Factors associated with diarrheal disease among under-five children

Table 4 presents the results of the logistic regression analysis on socioeconomic variables, like the number, age, and sex of the children, the educational and occupational status of the mothers, and the economic status of the households. The occurrence of diarrheal disease was associated with the number and age of under-five children in the households. The occurrence of diarrhea was 4.3 times more likely to be higher among households with two children compared with households with only one child [AOR = 4.3, 95% CI = (2.9, 6.3)]. Similarly, the likelihood of diarrhea occurrence was also 22.4 times higher among households with three children compared with households who had one child [AOR = 22.4, 95% CI = (7.8, 64.5)]. Children aged between 6.0 and 11.0 months had 4.8 times more chance to have diarrhea than children aged under 6 months [AOR = 4.8, 95% CI = (2.1, 10.8)]. Similarly, the occurrence of diarrhea among under-five children aged between 12.0–23.0 and 24.0–35.0 months was 6.0 and 2.5 times more likely to be higher compared with children aged under 6 months [AOR = 6.0, 95% CI = (2.9, 12.2)] and [AOR = 2.5, 95% CI = (1.2, 5.4)], respectively.

**Table 4:** Socioeconomic factors associated with childhood diarrhea among under-five children in Hadaleala District, Afar Region, northeast Ethiopia, May, 2015

Socioeconomic variables	Under-five diarrhea		COR (95% CI)	AOR (95% CI)
	Yes	No		
Number of children				
One	58	367	1	
Two	105	148	4.5 (3.1, 6.5)	4.3 (2.9, 6.3)**
Three	21	5	26.6 (9.6, 73.3)	22.4 (7.8, 64.5)**
Age of children in months				
<6.0	12	101	1	
6.0–11.0	28	44	5.4 (2.5, 11.5)	4.8 (2.1, 10.8)**
12.0–23.0	69	83	6.0 (3.6, 13.8)	6.0 (2.9, 12.2)**
24.0–35.0	31	107	2.4 (1.2, 5.0)	2.5 (1.2, 5.4)*
>35.0	44	185	2.0 (1.0, 4.0)	1.8 (0.9, 3.7)
Sex of children				
Male	89	289	0.8 (0.5, 1.1)	0.8 (0.5, 1.1)
Female	95	231	1	
Mothers’ education				
No formal education	174	450	2.7 (1.4, 5.4)	2.5 (1.2, 5.2)*
Formal education	10	70	1	
Household wealth status				
Poor	128	328	1.3 (0.9, 1.9)	1.6 (1.0, 2.4)*
Medium	56	192	1	

The result of Hosmer–Lemeshow test was >0.261

*Statistically significant variables at *p* < 0.05; **statistically significant variables at *p* < 0.001

Besides, childhood diarrheal disease was statistically associated with the educational status of mothers and household economic status. The likelihood of diarrhea occurrence was 2.5 times higher among children whose mothers had no formal education compared with their counterparts [AOR = 2.5, 95% CI = (1.2, 5.2)]. The occurrence of diarrhea was 1.6 times higher among children whose families were economically poor compared with children whose families had medium income [AOR = 1.6, 95% CI = (1.0, 2.2)].

## Discussion

This study investigated the prevalence of diarrheal disease and socioeconomic factors among under-five children of a nomadic community. The 2-week period prevalence of diarrheal disease among under-five children in Hadaleala District was 26.1% (95% CI 22.9, 29.3%). The finding of this study is slightly lower than the finding of a study conducted in Arba Minch District, 31.0% [9]. On the other hand, the finding of the current study is slightly higher than the findings of various studies conducted in the eastern part of Ethiopia, 22.5% [10], in a rural area of southern Ethiopia, 19.6% [19], in northwest Ethiopia, 24.9% [20], and Mecha District, 18.0% [5]. The high prevalence in the current study might be attributed to the difference in the socio- demographic, environmental, and behavioral characteristics of households and the nomadic nature of the population. As the communities living in the study area were nomads, they migrated from place to place in search of pasture and water. Having no permanent residential places, they may not have access to basic healthcare and sanitation services. Their main sources of water are rivers, streams, and wells which are prone to contamination. Because they have been practicing open defecation, their living environment is polluted with human excreta which is the main risk factor for diarrheal disease, especially for the children who routinely play in the unhygienic environment. Moreover, the people suffer from illiteracy and poverty which intern deteriorates their quality of life. All these phenomena are the direct risk factors for the occurrence of childhood diarrheal disease [7].

In this study, it was found that families who had two under-five children or above were more likely to have diarrhea than those who had only one child. As the number of children increased, the frequency of diarrhea increased significantly. This finding is supported by the findings of other similar studies. This can be justified by the fact that when the number of children in the household increases, it is expected that children could be more vulnerable to contamination because the quality of care and attention from parents decreases as mothers become incapable of caring for children. Furthermore, children who get diarrheal disease may easily transmit the disease to others who live in the same area [21–25].

The odds of having diarrheal morbidity were higher among children aged 6.0–11.0 and 12.0–23.0 months and lower at the age of 24.0 months and above compared to 0.0–5.0 months of age. The finding is consistent with that of other similar studies. This may be so because children aged more than 6 months start crawling or walking which increases their exposure to infectious agents. Moreover, such children start complementary feeding, and this may increase their exposure to different types of infections through contaminated food and water [5, 10, 22, 26–30].

This study indicated that maternal educational status was statistically associated with the occurrence of childhood diarrhea. Children whose mothers had attended formal education (primary and above) were less likely to develop diarrhea compared to children whose mothers had not attended any formal education. This may be due to the fact that education is likely to enhance household health and sanitation practices. Education can increase awareness about the transmission and prevention methods of diarrhea. It also encourages changes in behavior at the household level. Results of other studies agreed with this finding [8, 9, 31–34].

Household economic status was the other statistically associated variable. Children whose families were poor economically had higher odds of developing diarrhea compared with their counterparts. This may be due to the fact that rich families may have greater opportunity to use soap for hand washing and aqua-guard at their houses to protect microbial contamination in water and they may construct toilets. Lower income families were suffering from this disease because they could not afford these facilities [8, 11, 20, 28, 29, 33, 35, 36].

### Limitation of the study

Even though childhood diarrhea was properly defined by using the WHO diarrhea assessment tool, its occurrence was determined based on the reports of mothers without the confirmation of physicians. Due to this phenomenon, the study might be affected by social desirability bias. However, female data collectors who were part of the community were recruited owning to their strong relationships with mothers so they could minimize the social desirability bias. The other limitation of the study was the scarcity of literatures on nomads or a similar population; thus, the discussion was made on the basis of findings on the general population.

## Conclusions

The prevalence of diarrhea among under-five children in Hadaleala District was quite high. The highest rate of the prevalence was significantly concentrated among children aged 12.0–23.0 months. The childhood diarrheal disease was statistically associated with the number and age of under-five children, the educational level of mothers, and the economic status of households. To minimize the magnitude of childhood diarrheal disease, designing and implementing various prevention strategies, such as health education, child care, personal hygiene, and household sanitation, in integration with the existing national health extension program is recommended.

## Abbreviations

AOR: Adjusted odds ratio
CI: Confidence interval
COR: Crude odds ratio
EDHS: Ethiopian Demographic and Health Survey
IQR: Interquartile range
km: Kilometer
km^2^: Kilometer square
MOFED: Ministry of Finance and Economic Developments
SD: Standard deviation
SPSS: Statistical Package for Social Sciences
TLU: Tropical livestock holding

## References

[1] UNICEF. Levels and trends in child mortality, 2011 report. www.unicef.org/media/files/Child_Mortality_Report_2011_Final.pdf*.* Accessed 15 Sept 2016.

[2] PATH. Diarrheal disease: solutions to defeat a global killer. https://www.path.org/publications/files/IMM_solutions_global_killer_pp1-14.pdf. Accessed 16 Apr 2016.

[3] UNICEF. Water, sanitation and hygiene annual report 2013. www.unicef.org/…/WASH_Annual_Report_Final_7_2_Low_Res.pdf. Accessed 18 Apr 2016.

[4] Nyantekyi LA, Legesse M, Belay M, Tadesse K, Manaye K, Macias C, Erko B (2010). Intestinal parasitic infections among under-five children and maternal awareness about the infections in Shesha Kekele, Wondo Genet, Southern Ethiopia. Ethiop J Health Dev.

[5] Dessalegn M, Kumie A, Tefera W (2011). Predictors of under-five childhood diarrhea: Mecha District, West Gojam, Ethiopia. Ethiop J Health Dev.

[6] EDHS. Early childhood mortality rates by socioeconomic characteristics. Ethiopia Demographic and Health Survey, Central Statistical Agency Addis Ababa, Ethiopia, 2005. www.csa.gov.et/newcsaweb/images/…2005/…/DHS_survey_report_2005.pdf*.* Accessed 15 Mar 2016.

[7] Ethiopia. Hadaleala District. Finance and economic development office annual report 2014, by Dawud Haji Alisadik and others, Hadaleala: Officer of finance and economic development Afar Region, Ethiopia, 2014.

[8] Rahman A (2006). Assessing income-wise household environmental conditions and disease profile in urban areas: study of an Indian city. Geo J.

[9] Shikur M, Marelign T, Dessalegn T (2013). Morbidity and associated factors of diarrheal diseases among under five children in Arba-Minch District, Southern Ethiopia. Sci J Public Health.

[10] Mengistie B, Berhane Y, Worku A (2013). Prevalence of diarrhea and associated risk factors among children under-five years of age in eastern Ethiopia: a cross-sectional study. Open J Prev Med.

[11] Teklemariam S, Getaneh T, Bekele F (2000). Environmental determinants of diarrheal morbidity in under-five children, Keffa-Sheka zone, south west Ethiopia. Ethiop Med J.

[12] Mediratta PR, Feleke A, Moulton HL, Yifru S, Sack BR (2010). Risk factors and case management of acute diarrhoea in North Gondar zone, Ethiopia. J Health Popul Nutr.

[13] Mekasha A, Tesfahun A (2003). Determinants of diarrhoeal diseases: a community based study in urban south western Ethiopia. East Afr Med J.

[14] Green S, Small J, Casman A (2009). Determinants of national diarrhoeal disease burden. Environ Sci Technol.

[15] UNICEF/WHO. Diarrhoea: why children are still dying and what can be done. The United Nations Children’s Fund/World Health Organization, Geneva, 2009. www.unicef.org/…/Final_Diarrhoea_Report_October_2009_final.pdf*.* Accessed 18 May 2016.

[16] Black RE, Morris SS, Bryce J (2003). Where and why are 10 million children dying every year?. Lancet.

[17] Jahnke HE. Livestock production systems and livestock development in tropical Africa; livestock population in tropical Africa by species in numbers and in tropical livestock units (TLU) 1979. P. 10. www. pdf.usaid.gov/pdf_docs/PNAAN484.pdf. Accessed 10 May 2016.

[18] Grandin BE, Bekure S, Nestel P. Livestock transactions, food consumption and household budgets. FAO Corporate, Documentary Repository. http://www.fao.org/wairdocs/ILRI/x5552E/x5552e0a.htm. Accessed 3 Sept 2016.

[19] Tamiso A, Yitayal Y, Awoke A (2013). Prevalence and determinants of childhood diarrhoea among graduated households, in rural area of Shebedino District, Southern Ethiopia. Sci J Public Health.

[20] Gedefaw M, Takele M, Aychiluhem M, Tarekegn M. Current status and predictors of diarrhoeal diseases among under-five children in a rapidly growing urban setting: the case of city administration of Bahir Dar, northwest Ethiopia. Open J Epidemiol. 2015;5:89–97.

[21] Godana W, Mengistie B (2013). Determinants of acute diarrhoea among children under five years of age in Derashe District, Southern Ethiopia. Epub Rural Remote Health.

[22] Mihrete TS, Alemie GA, Teferra AS (2014). Determinants of childhood diarrhea among underfive children in Benishangul Gumuz Regional State, North West Ethiopia. BMC Pediatr.

[23] El-Gilany AH, Hammad S (2005). Epidemiology of diarrhoeal diseases among children under age 5 years in Dakahlia, Egypt. East Mediterr Health J.

[24] Shah MS, Yousafzai M, Lakhani BN, Chotanp AR, Nowshad G (2003). Prevalence and correlates of diarrhea. Indian J Pediatr.

[25] Arif A, Naheed R (2012). Socio-economic determinants of diarrhoea morbidity in Pakistan. Acad Res Int.

[26] Victor R, Baines SK, Agho KE, Dibley MJ (2013). Determinants of breastfeeding indicators among children less than 24 months of age in Tanzania: a secondary analysis of the 2010 Tanzania Demographic and Health Survey. BMJ Open.

[27] Calistus W, Alessio P (2009). Factors associated with diarrhea among children less than 5 years old in Thailand: a secondary analysis of Thailand multiple indicator cluster survey. J Health Res.

[28] Woldemicael G (2001). Diarrheal morbidity among children in Eritrea: environmental and socio-economic determinants. J Health Popul Nutr.

[29] Boadi KO, Kuitunen M. Childhood diarrheal morbidity in the Accra Metropolitan Area, Ghana: socio-economic, environmental and behavioral risk determinants. Journal of Health & Population in Developing Countries. 2005. http://www.jhpdc.unc.edu. Accessed 3 July 2016.

[30] Dewey KG, Adu-Afarwuah S (2008). Systematic review of the efficacy and effectiveness of complementary feeding interventions in developing countries. Matern Child Nutr.

[31] Anteneh A, Kumie A (2010). Assessment of the impact of latrine utilization on diarrheal diseases in the rural community of Hulet Ejju Enessie Woreda, East Gojjam Zone, Amhara Region. Ethiop J Health Dev.

[32] Yilgwan C, Yilgwan G, Abok I (2005). Domestic water sourcing and the risk of diarrhea: a cross-sectional survey of a semi-urban community in Nigeria. J Med.

[33] Gebru T, Taha M, Kassahun W (2014). Risk factors of diarrheal disease in under-five children among health extension model and non-model families in Sheko District rural community, Southwest Ethiopia: comparative cross-sectional study. BMC Public Health.

[34] Yilgwan CS, Okolo SN (2012). Prevalence of diarrhea disease and risk factors in Jos University Teaching Hospital, Nigeria. Ann Afr Med.

[35] Siziya S, Muula AS, Rudatsikira E (2009). Diarrhoea and acute respiratory infections prevalence and risk factors among under-five children in Iraq in 2000. Indian J Pediatr.

[36] Root GM (2001). Sanitation, community environments and childhood diarrhoea in rural Zimbabwe. J Health Popul Nutr.

